# Text message reminders and peer education increase HIV and Syphilis testing among female sex workers: a pilot quasi-experimental study in Uganda

**DOI:** 10.1186/s12913-021-06461-w

**Published:** 2021-05-07

**Authors:** Richard Muhindo, Andrew Mujugira, Barbara Castelnuovo, Nelson K. Sewankambo, Rosalind Parkes-Ratanshi, Juliet Kiguli, Nazarius Mbona Tumwesigye, Edith Nakku-Joloba

**Affiliations:** 1 Department of Nursing, College of Health Sciences, Makerere University, Kampala, Uganda; 2 Infectious Diseases Institute, College of Health Sciences, Makerere University, Kampala, Uganda; 3 School of Public Health, College of Health Sciences, Makerere University, Kampala, Uganda; 4 School of Medicine, College of Health Sciences, Makerere University, Kampala, Uganda; 5 Cambridge Institute of Public Health, University of Cambridge, Cambridge, United Kingdom

**Keywords:** HIV, Syphilis, Testing, Female Sex Workers, Africa, Text Messages

## Abstract

**Background:**

Globally, female sex workers (FSW) are disproportionately affected by HIV and other sexually transmitted infections (STIs). However, uptake of STI and HIV testing services among FSW in sub-Saharan Africa remains low. We aimed to assess the effect of FSW-led peer education and text message reminders on 3-monthly syphilis and HIV testing among FSW in Uganda.

**Methods:**

Between September 2019 and February 2020, we implemented weekly peer education sessions and bi-monthly SMS reminders for FSW in Mbarara (intervention city). Peer education sessions were implemented by 20 FSW, who received five days of basic training as peer educators. We held monthly meetings with peer educators throughout the six-month implementation period. FSW in Mbale (control city) continued to receive standard of care consisting of HIV testing outreach campaigns, and facility-based testing. Using a quasi-experimental design in one intervention city, and one control city, we conducted pre- and post- questionnaire-based surveys on recent syphilis and HIV testing behavior among FSW in July-October 2018, and March 2020. We compared proportions and prevalence ratios at baseline and follow-up using chi-square tests and negative binomial regression.

**Results:**

We conducted 436 interviews (200 before/236 after) with FSW. At baseline similar proportions reported taking an HIV test (57 % vs. 54 %; *p* = 0.72), and a syphilis serology test (35 % vs. 39 %; *p* = 0.67) in the intervention and control cities, respectively, in the prior three months. After the intervention, this proportion increased to 82 % (95 % confidence interval [CI] 74.0-88.2) for HIV, and 81 % (95 % CI: 73.0–87.0) for syphilis in the intervention city. Relative to baseline in the control city, the proportion testing for HIV was unchanged (52 %) but decreased for syphilis (26 %).

**Conclusions:**

Bi-monthly text message reminders with weekly peer education sessions increased uptake of 3-monthly syphilis and HIV testing in a Ugandan female sex work population and could help increase sex worker engagement in HIV/STI services in line with World Health Organization recommendations.

**Supplementary Information:**

The online version contains supplementary material available at 10.1186/s12913-021-06461-w.

## Introduction

Female sex workers (FSW) have a 30-times greater risk of human immunodeficiency virus (HIV) acquisition than women in the general population and are at increased risk of acquiring sexually transmitted infections (STIs) [1]. FSW contribute significantly to the annual global burden of 376 million new cases of curable STIs - syphilis, chlamydia, trichomoniasis and gonorrhoeae [2, 3]. In 2018, the median syphilis seroprevalence among FSW in sub-Saharan Africa (13.2 %) was four times higher than for FSW globally (3.2 %) [3]. The World Health Organization (WHO) recommends that FSW test for STIs like syphilis every 3–6 months, and for HIV every 6–12 months [4–6]. Ulcerative STIs - syphilis, gonorrhoeae, and herpes simplex virus type 2 (HSV-2) - increase HIV acquisition and transmission risk [7–9]. Prevention and treatment of HIV and other STIs has important personal and public health benefits; uptake of regular STI and HIV screening services is an important entry point into treatment, care and prevention [10, 11] and immediate treatment with antiretroviral therapy (ART) reduces HIV transmission risk by 93 % [12].

Studies conducted in Kampala, Uganda, show high HIV burden among FSW (30–52 % prevalence) [9, 13]. Factors associated with HIV positivity among FSW included never testing for HIV, self-reported genital ulcers and testing positive for STIs [9, 14]. Only 53–55 % of FSW reported having ever testing for HIV and only 16 % had received HIV testing in the preceding 12 months [9, 14]. Other work has reported high STI prevalence among FSW in Uganda: HSV-2 (80 %), trichomoniasis (17 %), gonorrhoeae (13 %), syphilis (10 %), and chlamydia (9 %) [9, 14, 15]. Effective STI control among key populations is associated with a decline in STI incidence in the general population, and syphilis seroprevalence among FSW is an important proxy indicator of progress in STI control [16]. However, uptake of STI and HIV testing services among FSW in sub-Saharan Africa remains low [17].

Mobile health (mHealth), and peer education interventions have been demonstrated to promote utilization of facility based STI services [18–20]. mHealth interventions in the form of text message reminders are more efficient in increasing rates of frequent HIV testing than community-based testing and self-testing in settings outside Africa [21]. In Uganda, community-based testing through moonlight outreach campaigns to key populations is the primary approach for promoting STI and HIV screening among FSW [22, 23]. However, this approach is not well utilized by FSW due to the limited services offered (HIV testing, health education, and condom distribution), and lack of privacy and confidentiality [24, 25]. Text message reminders offer an opportunity to promote frequent (i.e., more than yearly) HIV testing in line with WHO guidance for HIV and other STI screening. While evidence suggests text message reminders can be an effective strategy to reach people, link them to services and achieve behavioral change [26], promote ART adherence, retention in HIV care, and cervical cancer screening [27, 28], few studies in sub-Saharan Africa have evaluated their effect on FSW uptake of HIV/STI services. In Kenya, a recent study showed that using text message reminders to inform FSW about the availability of HIV self-testing at clinics increased HIV testing [29]. However, no study in Uganda has documented their effect on uptake of regular STI testing. Similarly, peer education interventions increased HIV testing (adjusted odds ratio [AOR] 4.9; 95 % CI: 2.8, 6.7) and STI examination and/or treatment ([AOR] 5.1; 95 % CI: 4.2, 6.4) among FSW in China [30]. Similar results were observed in India, Peru, and Benin [20, 31, 32] but little is known about how this mHealth intervention compares with peer education. In this study, we aimed to assess the effect of peer education and text message reminders on uptake of regular syphilis and HIV screening services among FSW in Uganda.

## Methods and materials

### Study site

The study was conducted in the cities of Mbale and Mbarara (combined general population 288,175) in Uganda [33]. Mbarara (Western Uganda) was the intervention city and Mbale (Eastern Uganda) was the control city. The two cities are located approximately 491 km apart and were chosen to minimize contamination (use of the intervention by control group members).

### Study design

This was a nonrandomized pilot quasi-experimental study with a pre- and post-measurement surveys. The study consisted of two phases. Prior to the intervention, we assessed intentions and frequency of recent syphilis and HIV testing among FSW in the cities of Arua, Mbale, Mbarara and Kampala (capital city), between July and October 2018 as previously described [34]. In the second phase, we implemented the intervention from September 2019 to February 2020. We assessed the effect of the intervention on uptake of regular syphilis and HIV among FSW in March 2020.

### Intervention Delivery

**Fig. 1:**
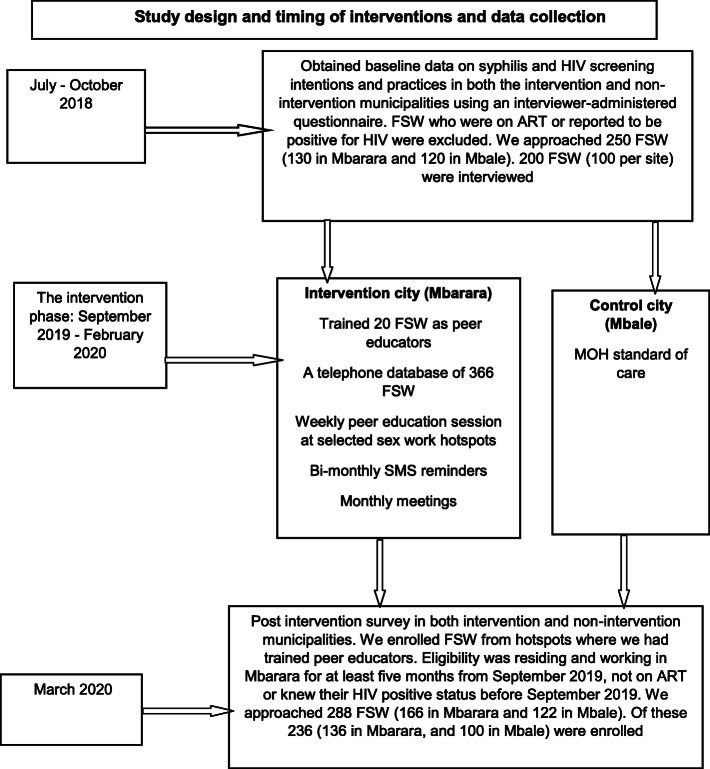
Study design and timing of interventions and data collection

First, we mapped out high volume sex work hotspots (locations frequented by sex workers where sex work occurs, e.g., bars, clubs, taverns, brothels, motels, hotels, and truck stops) [34]. Through consultation with local health workers and bar maids in Mbarara, we identified and trained 20 FSW as peer educators; in this setting, bar maids often double as sex workers. The peer educators received five days of training in basic knowledge about HIV and other STIs, key HIV/STI services for FSW, benefits of periodic HIV/STI testing, communication and counseling from a Ministry of Health (MoH) certified trainer. During training, the investigators, FSW peer educators, and the MoH trainer jointly designed the SMS reminder message, “*Feel proud to be tested for syphilis and HIV every three months. You are reminded to go and test today at the nearest health facility*”. Thereafter, the text message reminder was translated into Runyankore (local language). We built a database of telephone contacts through the FSW peer educators. During the solicitation of phone contacts, FSW peer educators informed FSW of the objectives of the study including the purpose for which their phone contact was being sought. FSW were also informed that their participation was voluntary. Before we implemented the text message reminder service, we verified ownership of FSW phone numbers through individual phone calls. We excluded FSW who had no phone of their own, did not know how to read text messages in English or Runyankore (Fig. 1). There is no charge for receiving a text message in Uganda.

Between September 2019 and February 2020, FSW peer educators delivered the intervention which consisted of weekly peer education sessions and bi-monthly text message (SMS) reminders. Content included educational messages on STIs, benefits of testing for syphilis and HIV every three months, and location of testing venues. Peer education sessions were conducted at sex work hotspots, and each FSW peer educator maintained a monthly record of their teaching sessions. Throughout the intervention period, we conducted monthly group meetings with the FSW peer educators in which we reviewed records and discussed any emerging challenges and potential solutions. Peer education sessions and bi-monthly SMS reminders were implemented concurrently. We aimed to reach FSW with both peer education and SMS reminders in Mbarara as a cluster. Standard of care HIV/STI testing services were provided to FSW in Mbale by Non-Governmental Organizations (NGOs), and the MoH through moonlight outreach campaigns and facility-based services [35–37]. In moonlight outreach campaigns, HIV prevention services (health education, counseling, testing, and condom distribution) are offered at selected sex work hotspots mainly at night.

### Data collection

Data collection was conducted both during the first phase from July - October 2018 (baseline), and in March 2020 (end-line) at follow-up in both the intervention and control cities, using an interviewer-administered questionnaire. Study participants included women aged ≥ 17 years and engaged in sex work according to self-report of selling sex for goods or money for at least 6 months, working [38] at a sex work hotspot with a trained peer educator and living in Mbarara for at least five months from September 2019 at end-line. At follow-up, data collectors who were not involved at baseline collected data in the intervention city. We administered questionnaires to collect data on syphilis and HIV screening intentions and practices; 200 before the intervention and 236 after intervention delivery. Printed HIV/STI result slips were provided to clients by testing facilities. We reviewed these HIV and syphilis test results for consenting respondents in the intervention city at follow-up and took care to preserve confidentiality. Respondents who reported receiving the intervention were asked to indicate on a 6-point Likert scale (1 = ‘strongly disagree’ to 6 = strongly agree’) how peer education or SMS reminders influenced their testing behaviors. FSW were asked to recite the text message and state the timing of the month when they received the bi-monthly SMS reminder.

### Recruitment procedures

We used a two-stage sampling design to recruit respondents at both baseline and follow-up, as previously described [34]. At follow-up, we purposely selected 20 sex work hotspots that had trained peer educators in the intervention city or had previously been sampled at baseline in the control city. We enrolled 2–3 FSW at each sex work hotspot through bar maids, with subsequent recruitment through peer referrals [34]. Referrals were made through a coupon containing the identification number, contact information, and duration of the survey in the city. Only participants who presented the coupon after verification, and met the eligibility criteria, were asked to provide informed consent to participate in an interviewer-administered questionnaire. The questionnaire was administered by trained research assistants at a venue agreed upon with the FSW. Follow-up visits to review HIV/STI test result slips for consenting respondents were conducted after the questionnaire interview in the intervention city. All study participants received information about STI and HIV screening during the consenting process and after the interview.

### Data analysis

Analyses were performed using Stata version 12.0 (StataCorp, College Station, TX). The primary outcome was syphilis and HIV testing in the prior three months. Respondents were asked if they had tested for syphilis and HIV and their readiness to take a syphilis serology test in the next 3 months and an HIV test in the next 6 months. We compared the proportions of FSW reporting testing for syphilis and HIV in the prior three months before and after in the intervention and control cities using chi-square statistics. Baseline and follow up data were analysed separately because we sampled different respondents at baseline and end-line. Baseline findings of the two cities combined were previously reported [34]. Proportions and prevalence ratios were used to describe the effect of the intervention (weekly peer education sessions and bi-monthly text message reminders) on recent (i.e., ≤ 6 month) syphilis and HIV testing, and intentions to take a syphilis serology test during the next three months and an HIV test during the next six months. Descriptive statistics were generated separately for the intervention and non-intervention cities at each of the data collection rounds (before and after) and are presented as proportions and p-values. To adjust for the potential effect of demographic characteristics (age, level of education, marital status and number of children), we compared the prevalence ratios (*P* < 0.05) in intervention and non-intervention cities using negative binomial regression (before and after).

### Ethical approval

Ethical clearance for the study was obtained from the Higher Degrees, Research and Ethics Committee, School of Public Health, Makerere University and Uganda National Council for Science and Technology (HS 2403). All respondents provided written informed consent in English or their local language. To protect human subjects, all members of the research team completed an online research ethics course with emphasis on research with vulnerable populations. FSW aged 17 years were included as emancipated minors in accordance with national guidelines [38]. All interviews were conducted at places agreed with participants where conversations could not be overheard, and no personal identifiers were obtained. No monetary payments were made to pimps and lodge/bar managers in order to avoid coercion in recruitment.

## Results

### Socio-demographic characteristics

A total of 436 (200 before/236 after) FSW were included in the analysis. We included 100 FSW per site at baseline, and 136 and 100 from Mbarara and Mbale, respectively, at end-line. Baseline and end-line survey respondents were generally similar with some differences in average number of children, clients per week, and marital status (Table 1). The median age was 26 years (interquartile range [IQR] 21–31), and 26 years (IQR 22–30) before and after, respectively in Mbarara compared to 28 years (IQR 24–32), and 27 years (IQR 23–31) in Mbale. The median duration of sex work before and after the intervention was 36 and 36 months in Mbarara relative to 40 and 38 months, respectively in Mbale. On average, FSW in Mbale reported more clients per week than in Mbarara (median of 26 and 25 compared to 17 and 15 clients before and after, respectively). At both baseline and end-line, the majority of FSW (85 % vs. 70 % in Mbale, and 77 % vs. 82 % in Mbarara) were mothers who solicited their clients on the street and in lodges, clubs and bars. Most of the FSW had obtained primary or no formal education (71 % vs. 70 % in Mbale, and 66 % vs. 64 % in Mbarara) before and after.

**Table 1:** Sex Worker Characteristics

Variable	Before			After		
	Intervention city *n* = 100	Control city *n* = 100	*p*-value	Intervention city *n* = 136	Control city *n* = 100	*p*-value
Age in years, median (IQR)	26 (21–31)	28 (24–32)	0.62	26 (22–30)	27 (23–31)	0.73
Duration of sex worker (months)	37 (19–68)	40 (24–72)	0.21	36 (18–66)	38 (23–70)	0.3
Clients per week, median (IQR)	17 (10–34)	26 (19–30)	0.01	15 (8–35)	25 (20–31)	0.01
Number of children, median (IQR)	1 (1–2)	2 (1–2)	0.03	2 (1–2)	1 (1–3)	0.02
**Education, n (%)**			0.89			0.2
None	16 (16.0)	16 (16.0)		20 (14.7)	15 (15.0)	
Primary	50 (50.0)	55 (55.0)		67 (49.3)	55 (55.0)	
Secondary	33 (33.3)	28 (28.0)		45 (33.1)	35 (35.0)	
Tertiary	1 (1.00)	1 (1.00)		4 (2.9)	0	
**Marital status, n (%)**			0.01			0.01
Married	1 (1.00)	10 (10.00)		4 (2.9)	2 (2.0)	
Separated	41 (41.0)	48 (48.0)		74 (54.4)	31 (31.0)	
Widow	5 (5.00)	10 (10.0)		1 (0.70)	6 (6.00)	
Single	53 (53.0)	32 (32.0)		57 (42.0)	61 (61.0)	
**Typology by venue, n (%)**						
Street	68 (33.0)	90 (45.5)	N/A	52 (25.0)	87 (43.0)	N/A
Home	1 (0.50)	0 (0.00)		2 (1.00)	1 (0.50)	
Lodge	87 (42.0)	76 (38.4)		62 (30.0)	84 (42.0)	
Bar/club	51 (24.6)	31 (15.7)		89 (43.0)	24 (12.0)	
Escort	0 (0.00)	1 (0.5)		2 (1.0)	5 (2.5)	

Baseline and end-line FSW cohorts were not the same

### Intervention Uptake and Testing Behaviors

In Mbarara, 101 respondents (74 %) received at least one intervention, 76 (75 %) received both peer education and SMS reminders, 15 (15 %) received peer education only, and 10 (10 %) received text message reminders (Table 2). Notably, seven FSW in Mbale received the intervention because of in-migration to the control city. Following receipt of both peer education and SMS reminders, 70 (92 %) and 72 (95 %) tested for syphilis and HIV, respectively, between September 2019 and February 2020. Of the respondents who received peer education, 88 (97 %) and 82 (90 %) tested for syphilis and HIV, respectively. Similarly, among those who received SMS reminders, 82 (93 %) and 77 (90 %) tested for syphilis and HIV during the same time period. Compared to SMS reminders, a slightly higher proportion of FSW reported being influenced by peer educators to test for syphilis (63.6 % vs. 57.3 %; *p* = 0.001) or HIV (48.2 % vs. 39.5 %; *p* = 0.001).

**Table 2:** Intervention uptake and testing behaviors

	Intervention city	Control city
**Variable**	**N (%)**	**N (%)**
**Received PE or SMS or both (Mbarara, *n* = 136, and Mbale*, n* = 100)**	**101 (74.2)**	**7 (7)**^a^
**Received PE (Mbarara, ***n*** = 101, and Mbale, *n* = 5)**	**91 (90)**	**2 (29)**
**Received SMS (Mbarara, *n* = 101, and Mbale, *n* = 5)**	**86 (85)**	**4 (57)**
**Received both PE and SMS (Mbarara, *n* = 101)**	**76 (75.2)**	**1 (14)**
**Received PE only (Mbarara, n = 01)**	**15 (14.9)**	**NA**
**Received SMS only (Mbarara, *n* = 101)**	**10 (9.9)**	**NA**
**Received PE and tested for syphilis (Mbarara, *n* = 91)**	88 (96.7)	NA
**Received phone SMS and tested for syphilis (Mbarara, *n* = 86)**	82 (93.2)	NA
**Received both PE and TM, and tested for syphilis (Mbarara, *n* = 76)**	70 (92.0)	NA
**Btn Sept 2019 and Feb, 2020, PE influenced your syphilis testing behavior (Mbarara, *n* = 91)**		
Strongly agree/agree	56 (63.6)	NA
**Btn Sept 2019 and Feb 2020, SMS influenced your syphilis testing behavior (Mbarara, *n* = 86)**		
Strongly agree/agree	47 (57.3)	NA
**Anticipating to receive money influenced your syphilis testing behavior**		
Strongly agree/agree	3 (2.5)	NA
**Received PE and tested for HIV (Mbarara, *n* = 91)**		
Yes	82 (90.1)	NA
**Received SMS and tested for HIV (Mbarara, *n* = 86)**		
Yes	77 (89.5)	NA
**Received both PE and SMS, and tested for HIV (Mbarara, *n* = 76)**		
Yes	72 (95.0)	NA
**Btn Sept 2019 and Feb 2020, a peer educator influenced your HIV testing behaviour**		
Strongly agree/agree	40 (48.2)	NA
**Btn Sept 2019 and Feb 2020, a reminder SMS message on your phone influenced your HIV testing behaviour**		
Strongly agree/agree	30 (39.5)	NA
**Anticipating to receive money influenced your HIV testing behaviour**		
Strongly agree/agree	5 (4.6)	NA

^a^Owing to the mobility of FSW, a very small proportion in the control city received SMS reminders and peer education sessions, *SMS* text message reminder, *PE* peer education, NA,

### Intervention Effect on syphilis and HIV testing

At end-line in Mbarara, the proportion of FSW intending to take a serological syphilis test in the next three months was high (93 %) compared to 36 % at baseline, as was self-report of ever testing for syphilis (90 % vs. 50 %) (Table 3). Similiarly, 81 % of the FSW reported taking a syphilis serology test in the prior three months compared to 35 % at baseline. While more FSW (49 %) reported intention to take a syphilis serology test in the next three months compared to 37 % at baseline, no difference was observed (60 % vs. 61 %) in self-report of ever testing in Mbale. Furthermore, the proportion of FSW reporting testing in the prior three months was low (26 %) compared to 39 % at baseline. Despite observing no difference in the proportion of FSW reporting ever testing for HIV at baseline and endline in both Mbarara and Mbale, self-report of testing in the prior three months was high (82 %) in Mbarara compared to 57 % at baseline. Self-report of recent HIV testing in Mbale was similar (52 % vs. 54 %).

**Table 3:** Intervention effect on Syphilis and HIV testing

	Intervention city			Control city		
	Before	After	*p*-value	Before	After	*p*-value
	*N* = 100	*N *= 136		*N* = 100	*N* = 100	
	Proportion (95 % CI)			Proportion (95 % CI)		
Condom use at last sex (Yes)	0.76 (0.67-0.83)	0.73 (0.65-0.79)	0.49	0.88 (0.79-0.93)	0.91 (0.83-0.95)	0.74
Consistently use condoms (Yes)	0.64 (0.53 − 0.72)	0.33 (0.25–40)	**0.001**	0.61 (0.50- 0.70)	0.67 (0.57 − 0.76)	**0.045**
**Intention to test for syphilis in next 3 months**			**0.001**			**0.03**
High	0.36 (0.27-0.46)	0.93 (0.87-0.96)		0.37 (0.28-0.47)	0.49 (0.39-0.59)	
**Ever taken a serological syphilis test**			0.001			0.42
Yes	0.50 (0.40-0.59)	0.90 (0.84-0.94)		0.61 (0.51 − 0.70)	0.60 (0.50 − 0.70)	
**Recent serological syphilis testing**			**0.001**			**0.02**
Prior 3 months	0.35 (0.23-0.49)	0.81 (0.73-0.87)		0.39 (0.28 − 0.52)	0.26 (0.17-0.39)	
Prior 6 months	0.25 (0.15-0.39)	0.15 (0.10-0.23)		0.18 (0.10 − 0.30)	0.13 (0.07-0.24)	
Prior 12 months	0.18 (0.09-0.31)	0.02 (0.04-0.06)		0.20 (0.11 − 0.32)	0.26 (0.17-0.39)	
More than 12 months	0.22 (0.12-0.35)	0.02 (0.04-0.06)		0.23 (0.14 − 0.36)	0.34 (0.23-0.47)	
**Intention to test for HIV in the next 6 months**			**0.03**			0.44
High	0.88 (0.80 − 0.93)	0.99		0.83 (0.74 − 0.89)	0.87 (0.78-0.93)	
**Ever tested for HIV**			0.38			0.98
Yes	0.95 (0.88-0.97)	0.97 (0.92-0.99)		0.92 (0.86-0.97)	0.93 (0.85-0.96)	
**Recent HIV testing**			**0.001**			0.93
Prior 3 months	0.57 (0.47-0.67)	0.82 (0.74-0.88)		0.54 (0.43-0.64)	0.52 (0.42-0.63)	
Prior 6 months	0.27 (0.19 − 0.37)	0.11 (0.07-0.19)		0.30 (0.22-0.40)	0.33 (0.22-0.43)	
Prior 12 months	0.07 (0.04-0.15)	0.04 (0.01-0.09)		0.06 (0.03-0.14)	0.1 (0.05-0.19)	
More than 12 months	0.08 (0.04-0.16)	0.02 (0.01–08)		0.10 (0.05 − 0.17)	0.05 (0.02-0.11)	

### Factors associated with HIV and syphilis testing

In multivariate analysis after adjustment for age, level of education, marital status, and number of children, the effect of the intervention was an almost four-fold higher report of testing for syphilis in the prior three months in Mbarara relative to Mbale (adjusted prevalence ratio [aPR] 3.59, 95 % CI: 2.31–5.56) (Table 4).

**Table 4:** Factors associated with recent syphilis testing

	Before (2018)		After (2020)	
	Adjusted Prevalence Ratio (95 % CI)	*p*-value	Adjusted Prevalence Ratio (95 % CI)	*p*-value
**City**				
Control	Reference		Reference	
Intervention	0.84 (0.52–1.35)	0.47	3.59 (2.31–5.56)	**0.001**
Received SMS + Peer education				
No			Reference	
Yes			1.18 (0.86–1.61)	0.30
**Age (years)**				
17–24	Reference		Reference	
25–30	0.85 (0.47–1.53)	0.58	1.08 (0.75–1.56)	0.67
≥ 31	0.65 (0.32–1.34)	0.23	0.92 (0.59–1.41)	0.69
**Children**				
No	Reference		Reference	
Yes	1.67 (0.76–3.65)	0.19	1.14 (0.72–1.78)	0.58
**Education level**				
None	Reference		Reference	
Primary	0.96 (0.49–1.89)	0.92	1.10 (0.64–1.91)	0.65
Secondary	1.34 (0.66–2.72)	0.41	1.19 (0.68–2.11)	0.43
Tertiary	1.48 (0.17–12.80)	0.72	0.86 (0.38–1.92)	0.72
**Marital status**				
Married	Reference		Reference	
Separated	0.49 (0.24–1.04)	0.06	0.69 (0.32–1.49)	0.34
Widow	0.54 (0.19–1.48)	0.23	0.18 (0.02–1.54)	0.11
Single	0.18 (0.06–0.55)	0.003	0.59 (0.27–1.31)	0.19
Has boyfriend	0.48 (0.19–1.17)	0.11	0.68 (0.29–1.53)	0.34

In multivariate analysis after adjustment for age, level of education, marital status, and whether one had children, there was an increase of 19 % in the likelihood of reporting testing for HIV in the prior three months among FSW in Mbarara when compared to FSW in Mbale [aPR] 1.19, 95 % CI: 0.83–1.70), but this finding was not statistically significant. Relatedly, the likelihood of testing in the prior three months was lower among FSW who did not receive a text message reminder or peer education (28 % vs.17 %) when compared with FSW who received a text reminder or peer education [aPR] 0.72, 95 % CI: 0.51–1.02 and [aPR] 0.83, 95 % CI: 0.58–1.19) respectively (Table 5).

**Table 5:** Factors associated with recent HIV testing

	Before (2018)		After (2020)	
	Adjusted prevalence ratio (95 % CI)	*p*-value	Adjusted prevalence ratio (95 % CI	*p*-value
**City**				
control	Reference		Reference	
intervention	1.26 (0.77–2.09)	0.36	1.19 (0.83–1.70)	0.35
Received SMS reminder				
Yes			Reference	
No			0.72 (0.51–1.02)	0.07
Received peer education				
Yes			Reference	
No			0.83 (0.58–1.19)	0.35
**Age (years)**				
17–24	Reference		Reference	
25–30	1.04 (0.73–1.51)	0.84	1.16 (0.85–1.57)	0.32
≥ 31	0.83 (0.48–1.43)	0.50	1.04 (0.72–1.52)	0.80
**Children**				
No	Reference			
Yes	0.95 (0.64–1.43)	0.82	1.02 (0.74–1.41)	0.57
**Education level**				
None	Reference		Reference	
Primary	1.06 (0.68–1.65)	0.80	1.05 (0.66–1.68)	0.80
Secondary	1.20 (0.75–1.93)	0.44	1.17 (0.83–1.89)	0.49
Tertiary	0.57 (0.08–4.36)	0.59	1.24 (0.66–2.31)	0.49
**Marital status**				
Married	Reference		Reference	
Separated	0.96 (0.37–2.53)	0.06	1.18 (0.56–2.48)	0.67
Widow	0.71 (0.23–2.18)	0.55	1.32 (0.37–3.67)	0.58
Single	0.70 (0.25–1.95)	0.49	1.18 (0.56–2.48)	0.67
Has boyfriend	0.97 (0.36–2.57)	0.96	1.34 (0.62–2.88)	0.44

## Discussion

 In this pilot quasi-experimental study, in which we assessed the effect of peer education and text message reminders on intentions and uptake of syphilis and HIV testing among FSW, there was a three-fold increase in the proportion of FSW intending to take a syphilis serology test in the next three months and a two-fold increase in self-report of ever testing for syphilis in the prior three months in Mbarara, the intervention city, relative to baseline. Similarly, there was a significant increase in HIV testing in the prior three months. In contrast, not much difference was observed between the endeline and baseline data in Mbale, the control city.

Our finding that 3-monthly testing for syphilis and HIV significantly improved after the combined intervention suggests that mobile phone text message reminders and peer education can increase uptake of syphilis and HIV testing services. Similar findings have been reported by studies evaluating the effect of text message reminders on uptake of regular HIV testing [21]. However, most of these studies were conducted in setings outside Africa, and focused on HIV. A recent study in Kenya showed that that informing FSW about the availability HIV self-testing at clinics via text message reminders resulted in a 2-fold increase in HIV testing [29]. A systematic review found that peer education interventions are associated with a 3-fold increase in HIV testing with a consistent impact on behavioral change for over 24 months [39]. Peers act as positive role models and provide informational, emotional and affiliation support for utilization of testing services [40].

Little is known about how mHealth interventions compare with peer education. A higher proportion of FSW in our study reported being influenced by peer educators to test for syphilis. mHealth interventions have demonstrated positive impact in improving attendance rates at health facilities [41] but lack of emotional and affiliation support offered by peer educators may limit their efficacy. However, FSW go through different stages of behavior change [42, 43] and text message reminders may be ineffective at the pre-contemplation phase. Our findings add to the literature showing that text message reminders and peer education improve utilization of STI and HIV testing services [44].

Peer education and text messaging may reinforce each other [45]. Peer education may have improved knowledge of the benefits of periodic syphilis and HIV testing, while text messages acted to remind and reinforce information received during peer education. The resultant empowerment and self-efficacy may explain the increase in testing uptake that we observed [46]. Public health programs that combine both mHealth and peer education interventions may be more desirable for promoting regular syphilis and HIV testing among FSW.

The strengths of this study include the ability to describe the effect of both text message reminders and peer education; data describing how text message reminders compare with peer education are limited. Our study has limitations and care should be taken when interpreting and generalizing these results. We evaluated a single intervention and non-intervention city with a non-random sample which might limit the generalizability of our findings. We obtained phone numbers of FSW through trained peer educators recruited from selected sex work hotspots and as such our findings may not be representative of all FSW. We enrolled participants from the same sex hotspots where we recruited peer educators and the phone numbers we obtained may not be representative of all sex workers who received SMS reminders. A significant proportion of FSW had primary or no education and may not have been able to read text messages. Social desirability and recall bias may have influenced reporting of syphilis and HIV testing behaviours in Mbale, but not in Mbarara where we cross checked test result slips to ascertain self-reported syphilis and HIV test status for more than 80 % of respondents.

## Conclusions

HIV and STI control programs should utilize bi-monthly text message reminders with weekly peer education sessions to promote periodic testing for syphilis and HIV among FSW. Future research should evaluate whether combining text message reminders with peer education is more effective than either peer education or text message reminders alone.

The authors thank the leadership of the cities of Mbarara, and Mbale which enabled the data collection process. We thank the participants for volunteering to participate in the study. Finally, we thank Collins Twesigye, and Jonan Mweteise who worked as research assistants.

## Supplementary Information

**Additional file 1**

## Data Availability

The datasets used during the current study are available from the corresponding author on request. The questionnaire is included as supplementary information.

## Abbreviations

FSW: Female sex workers
HIV: Human immunodeficiency virus
IQR: Interquartile range
CI: Confidence interval
PSU: Primary sampling unit
STI: Sexually transmitted infection
SMS: Text messages
WHO: World Health Organization
