# Supplementary material for: Text message reminders and peer education increase HIV and Syphilis testing among female sex workers: a pilot quasi-experimental study in Uganda
Source: BMC Health Serv Res. 2021 May 7;21:436. doi: 10.1186/s12913-021-06461-w (PMC8103763; doi:10.1186/s12913-021-06461-w)
Supplement: Supplementary file 1 — Additional file 1 [file 12913_2021_6461_MOESM1_ESM.docx]

**Text Message Reminders and Peer Education Increase HIV and Syphilis Testing among Female Sex Workers: A Pilot Quasi-Experimental Study in Uganda**

Richard Muhindo^1^, Andrew Mujugira^2,3^, Barbara Castelnuovo^2^, Nelson K. Sewankambo^4^, Rosalind Parkes-Ratanshi^2,5^, Juliet Kiguli^3^, Nazarius Mbona Tumwesigye^3^ and Edith Nakku-Joloba^3^

1. Department of Nursing; 2. Infectious Diseases Institute; 3. School of Public Health and; 4. School of Medicine, College of Health Sciences, Makerere University, Kampala, Uganda; 5. Cambridge Institute of Public Health, University of Cambridge, Cambridge, United Kingdom

Correspondence: Richard Muhindo, Department of Nursing, College of Health Sciences, Makerere University

Email: [r.muhindo@yahoo.com](mailto:r.muhindo@yahoo.com)

**THE SURVEY QUESTIONNAIRE**

SECTION: SOCIODEMOGRAPHIC CHARACTERISTICS

| No. | Questions and filters | Answers and codes | Shift to |
| --- | --- | --- | --- |
| Q01 | Age in complete years |  |  |
| Q02 | Highest level of education attained; primary, secondary, higher education  *Interviewer, circle only one answer* | No education…………..0  Primary …………………….1  Secondary……………………2  Higher education……………..3 |  |
| Q03 | Marital status  *Interviewer, list all the possibilities. There can be more than one answer circled* | Married…………………..1  Separated………………….2  Widow…………………….3  Single………………………4  Have a boyfriend…………...5 |  |
| Q04 | Number (biological) of children currently |  |  |
| Q5 | Average number of clients per week |  |  |
| Q6 | Average amount charged per client |  |  |
| Q07 | Duration in sex work in completed months |  |  |
| Q08 | How would describe your current work  *Interviewer, list all the possibilities. There can be more than one answer circled* | Full time, as I have no other sources of income……………………….1  Part-time, as I have other sources of income……………………….2  Part-time, as I am a student……..3  Full-time, to supplement my other sources of income  Others specify………………………. |  |
| Q09 | Venue of operation  *Interviewer, list all the possibilities. There can be more than one answer circled* | Street ……………………………………..1  Home………………………………………2  Lodge……………………………………….3  Bar…………………………………………..4  Brothel………………………………………5  Others specific………………………………. |  |
| Q10 | Ever heard about PrEP | Yes ………………..1  NO………………….2 |  |
| Q11 | Ever Heard about PEP | Yes …………………1  No……………………2 |  |
| Q12 | PrEP use | I have never used PrEP……………….1  I have ever used PrEP………………….2  I am currently using PrEP………………3 |  |
| Q13 | PEP use | I have never used PEP………………..1  I have ever used PEP………………….2 |  |
| Q14 | Condom use at last sexual intercourse | Yes……………………….1  No………………………….2 |  |
| Q16 | Based on 10 clients, how many would you say you consistently use a condom |  |  |
| Q17 | How would you describe your condom use practice in your work  *Interviewer, many answers are possible* | I consistently use condom at every sexual intercourse with all my clients…………………………….1  I sometimes don’t use a condom with some clients………………….…………2 |  |
|  | Between October, 2019 and March, 2020, a female peer educators talked you about testing for syphilis and HIV | Yes ………………………………………1  No…………………………………………2 |  |
|  | Between October, 2019 and March, 2020, you received a reminder message on your phone about testing for syphilis and HIV | Yes……………………………………..1  No……………………………………….2 |  |

**SECTION; STI AND HIV TESTING BEHAVIOR**

| Q18 | Have you already gone to have a serological syphilis test  If yes, between October, 2019 and March 2020, how often did you a serological syphilis test done | Yes ……………………………1  No………………………………2  Never………………………….1  Once……………………………2  Twice…………………………..3  Three times…………………...4  Four times or more…………….5 |  |
| --- | --- | --- | --- |
| Q19 | Where (public health facility, private clinic, outreach) did you have the last screening done | Place; **________________________** |  |
| Q20 | Before October, 2019, how many times did you test for syphilis in 6 months? | Never………………………….1  Once……………………………2  Twice…………………………..3  Three times…………………...4  Four times or more…………….5 |  |
| Q21 | ***If you took a syphilis serological test between October, 2019 and March, 2020, on scale of 1 to 6, where 1 = strongly disagree and 6, strongly agree, how did the following motivate to go for the test*** | | |
|  | A peer educator | Strongly agree………………….1  Disagree…………….………….2  Somewhat disagree……………….3  Somewhat agree…………………………….4  Agree………….…………………5  Strongly agree……………………6 |  |
|  | An SMS message reminder on my phone | Strongly agree………………….1  Disagree…………….………….2  Somewhat disagree……………….3  Somewhat agree…………………………….4  Agree………….…………………5  Strongly agree……………………6 |  |
|  | Anticipation to receive money | Strongly agree………………….1  Disagree…………….………….2  Somewhat disagree……………….3  Somewhat agree…………………………….4  Agree………….…………………5  Strongly agree……………………6 |  |
|  | Others specify | …………………………………………….  …………………………………………….  …………………………………………….. |  |
| Q22 | If you don’t mind, what were the results | I was found positive for syphilis………1  I was found negative for syphilis ……...2 |  |
| Q24 | Have you already gone for an HIV test  If yes, between October, 2019 and March, 2020 how often did you test for HIV | Yes--------------------------------1  No---------------------------------2  Never-----------------------------1  Once------------------------------2  Twice-----------------------------3  Thrice-----------------------------4  Four or more…….…………….5 |  |
| Q25 | Where (public health facility, private clinic, outreach) did you have the last screening done | Place; **________________________** |  |
| Q26 | Before October, 2019, how many times did you test for HIV in 6 months | Never………………………….1  Once……………………………2  Twice…………………………..3  Three times…………………...4  Four times or more…………….5 |  |
|  | ***If you took an HIV test between October, 2019 and March, 2020, how did the following motivate you on a scale of 1 to 6 (1 = strongly disagree, 6 = strongly agree)*** | | |
| Q27 | A peer educator | Strongly agree………………….1  Disagree…………….………….2  Somewhat disagree……………….3  Somewhat agree…………………………….4  Agree………….…………………5  Strongly agree……………………6 |  |
|  | An SMS message reminder on my phone | Strongly agree………………….1  Disagree…………….………….2  Somewhat disagree……………….3  Somewhat agree…………………………….4  Agree………….…………………5  Strongly agree……………………6 |  |
|  | Anticipation to receive money | Strongly agree………………….1  Disagree…………….………….2  Somewhat disagree……………….3  Somewhat agree…………………………….4  Agree………….…………………5  Strongly agree……………………6 |  |
|  | Others specify | ……………………………………  …………………………………….  ……………………………………. |  |
| Q29 | If you don’t mind, what was the test result | I was found positive ………………….1  I went when I knew I was positive……2  I was found negative ………………….3 |  |
| Q30 | Preferences of where you would want to have an STI and HIV tests done. | Places;______________________ |  |

**SECTION; Major independent variables**

Introduction: This section is reserved for HIV negative people or those unware of their HIV or STI status.

The questions focus on the intention to go for STI screening in the next 3 to 6 months and HIV testing in the next 6 to 12 months.

**Interviewer**; advice participants to response to the best of their knowledge. Specify to them that there are no right or wrong answer. The most important thing is to say how the situation described apply to you. So please answer the question by saying what you really think.

Interviewer; Explain and demonstrate scale to the participant so that she can see and chose a response on the scale for each item.

**Section: intention**

| No. | Statement | Answers and codes |
| --- | --- | --- |
| To what extend do you agree or disagree with the following statements | | |
| Q31 | You intend to go for Syphilis screening during the next 3-6 months | Strongly disagree---------------1  Disagree------------------------2  Somewhat disagree------------3  Somewhat agree----------------4  Agree-----------------------------5  Strongly agree------------------6 |
| Q32 | You are going to be screened for syphilis during the next 3-6 months | Strongly disagree---------------1  Disagree------------------------2  Somewhat disagree------------3  Somewhat agree----------------4  Agree-----------------------------5  Strongly agree------------------6 |
| Q33 | How would you evaluate your chances that you would be screened for Syphilis in the next 3-6 months | Neither low nor high--------------1  Very low----------------------------2  Low----------------------------------3  Moderate----------------------------4  High---------------------------------5  Very high………………………6 |
| Q34 | You intend to go for HIV testing during the next 6-12 months | Strongly disagree---------------1  Disagree------------------------2  Somewhat disagree------------3  Somewhat agree----------------4  Agree-----------------------------5  Strongly agree------------------6 |
| Q35 | You are going to be tested for HIV during the next 6-12 months | Strongly disagree---------------1  Disagree------------------------2  Somewhat disagree------------3  Somewhat agree----------------4  Agree-----------------------------5  Strongly agree------------------6 |
| Q36 | How would you evaluate your chances that you would be tested for HIV in the next 6-12 months | Neither low nor high--------------1  Very low----------------------------2  Low----------------------------------3  Moderate----------------------------4  High---------------------------------5  Very high………………………6 |
| 37 | If you do not intent to go a Syphilis checkup in the next 3-6 months, kindly what are your reasons? *Interviewer; list all mentioned* |  |
| 38 | If you do not intent to go an HIV test in the next 6-12 months, kindly what are your reasons?  *Interviewer; list all mentioned* |  |

This is the end of our questionnaire. Thank you very much for your time in answering these questions.

We appreciate your assistance.
